# Antimicrobial resistance in *Mycoplasma genitalium* sampled from the British general population

**DOI:** 10.1136/sextrans-2019-054129

**Published:** 2020-01-10

**Authors:** Rachel Pitt, Magnus Unemo, Pam Sonnenberg, Sarah Alexander, Simon Beddows, Michelle Jayne Cole, Soazig Clifton, Catherine H Mercer, Anne M Johnson, Catherine A Ison, Nigel Field

**Affiliations:** 1 National Infection Service, Public Health England, London, United Kingdom; 2 WHO Collaborating Centre for Gonorrhoea and Other STIs, National Reference Laboratory for STIs, Department of Laboratory Medicine, Microbiology, Faculty of Medicine and Health, Örebro University, Orebro, Sweden; 3 Centre for Population Research in Sexual Health and HIV, Institute for Global Health, UCL, London, United Kingdom; 4 Centre for Molecular Epidemiology and Translational Research, Institute for Global Health, UCL, London, United Kingdom

**Keywords:** mycoplasma, antibiotic resistance, molecular epidemiology, public health, Mycoplasma genitalium

## Abstract

**Background:**

*Mycoplasma genitalium* is a common sexually transmitted infection. Treatment guidelines focus on those with symptoms and sexual contacts, generally with regimens including doxycycline and/or azithromycin as first-line and moxifloxacin as second-line treatment. We investigated the prevalence of antimicrobial resistance (AMR)-conferring mutations in *M. genitalium* among the sexually-active British general population.

**Methods:**

The third national survey of sexual attitudes and lifestyles (Natsal-3) is a probability sample survey of 15 162 men and women aged 16–74 years in Britain conducted during 2010–12. Urine test results for *M. genitalium* were available for 4507 participants aged 16–44 years reporting >1 lifetime sexual partner. In this study, we sequenced regions of the 23S rRNA and *parC* genes to detect known genotypic determinants for resistance to macrolides and fluoroquinolones respectively.

**Results:**

94% (66/70) of specimens were re-confirmed as *M. genitalium* positive, with successful sequencing in 85% (56/66) for 23S rRNA and 92% (61/66) for *parC* genes. Mutations in 23S rRNA gene (position A2058/A2059) were detected in 16.1% (95%CI: 8.6% to 27.8%) and in *parC* (encoding *ParC* D87N/D87Y) in 3.3% (0.9%–11.2%). Macrolide resistance was more likely in participants reporting STI diagnoses (past 5 years) (44.4% (18.9%–73.3%) vs 10.6% (4.6%–22.6%); p=0.029) or sexual health clinic attendance (past year) (43.8% (23.1%–66.8%) vs 5.0% (1.4%–16.5%); p=0.001). All 11 participants with AMR-conferring mutations had attended sexual health clinics (past 5 years), but none reported recent symptoms.

**Conclusions:**

This study highlights challenges in *M. genitalium* management and control. Macrolide resistance was present in one in six specimens from the general population in 2010–2012, but no participants with AMR *M. genitalium* reported symptoms. Given anticipated increases in diagnostic testing, new strategies including novel antimicrobials, AMR-guided therapy, and surveillance of AMR and treatment failure are recommended.

## Introduction

*Mycoplasma genitalium* is a widespread STI causing urogenital infection in men and women.^1–3^ *M. genitalium* infection is a frequent cause of non-gonococcal urethritis in men, and is associated with cervicitis, pelvic inflammatory disease, preterm delivery, spontaneous abortion, and infertility in women.^2–6^ Most individuals with *M. genitalium* are asymptomatic, and the clinical implications of symptomless infection remain unclear.

In the British general population, in 2010–2012, the urogenital prevalence of *M. genitalium* was similar to that of *Chlamydia trachomatis* in those aged 16–44 years old, although co-infection was rare. In men, *M. genitalium* prevalence was 1.2% (95% CI 0.7% to 1.8%) compared with *C. trachomatis* prevalence of 1.1% (0.7%–1.6%), and in women was 1.3% (0.9%–1.9%) compared with 1.5% (1.1%–2.0%) respectively.^7 8^ Higher *M. genitalium* prevalence has been observed in men who have sex with men (MSM) (3.2% (2.1%–5.1%)) and female sex workers (15.9% (13.5%–18.9%)).^1^

National guidelines for *M. genitalium* are lacking or only recently introduced for many countries.^9^ Existing international and national guidelines, including the 2018 UK national guidelines, focus on testing those with symptoms and sexual contacts of cases, citing a lack of evidence to recommend routine screening in asymptomatic individuals, which might have implications for selection of antimicrobial resistance (AMR).^9–11^ Due to suboptimal diagnostics and a lack of testing even within specialist centres, *M. genitalium* is likely to be widely underdiagnosed.

The fastidious nature and slow growth of the organism makes culture challenging, time-consuming, and unsuitable for routine diagnostic testing. Accordingly, routine AMR phenotyping of *M. genitalium* is not feasible, and AMR-testing currently relies on the detection of genotypic changes within genomic loci associated with phenotypic resistance and treatment failure. Current treatment guidelines typically recommend a macrolide (azithromycin) or tetracycline (doxycycline) followed by azithromycin, ideally after AMR testing, as first line and a fluoroquinolone (moxifloxacin) as second line treatment.^9–11^ However, azithromycin resistance (5%–100%) and moxifloxacin resistance (0%–30%) have been reported in studies from Europe, Australia, New Zealand, Japan, and USA.^12–15^ These data derive from clinic-based studies, and there are currently no data on the occurrence of *M. genitalium* AMR determinants in specimens from the general population to inform policy decisions.

Taken together, *M. genitalium* is an important and prevalent STI, and one where management and control is challenging because it is under-tested, under-detected, and difficult to treat.^16^ Moreover, increasing availability of diagnostic tests is likely to increase opportunities for screening asymptomatic patients, but the risks and benefits need careful consideration.^17^ This situation requires a considered strategic public health response. We investigated the distribution of genotypically determined resistance in *M. genitalium* positive specimens from the sexually-active British general population.

## Methods

### Participants and survey procedures

The third National Survey of Sexual Attitudes and Lifestyles (Natsal-3) was a stratified probability sample survey of 15 162 men and women in Britain (England, Scotland, and Wales) aged 16–74 years who were interviewed in 2010–2012. The estimated overall response rate was 57.7% and the cooperation rate was 65.8% (of all eligible addresses contacted).^18^ Participants were interviewed using computer-assisted face-to-face and self-completion interviews (CASI); further methodological details have been described elsewhere.^19^ The CASI included questions about participants' sexual behaviour, sexual health clinic attendance, current STI symptoms, and history of being diagnosed with STIs by a healthcare professional. After the interview, we invited a sample of participants aged 16–44 years to provide urine for STI testing.^8^ First-void urine (4–5 mL) was collected with the FirstBurst device and posted to Public Health England for testing. All participants were provided with information on where to obtain free diagnostic STI/HIV testing and sexual health advice.^19^

### Laboratory methods

Urine specimens were tested using an in-house real-time PCR (RT-PCR) assay which targets the *M. genitalium* adhesin protein (*MgPa*) gene (assay modified from Jensen *et al*. 2004),^20^ with positive or equivocal results confirmed using a research-use-only Aptima *M. genitalium* assay (Hologic Inc., San Diego, USA). Urine test results were available for *M. genitalium* on 4507 specimens and 72 were positive. For specimens where participants had provided consent for storage (70/72), the presence of *M. genitalium* DNA was re-confirmed in the present study using the in-house *MgPa* RT-PCR.^20^ Macrolide and fluoroquinolone resistance determinants were assessed by PCR and sequencing of resistance-associated regions of the 23S rRNA and *parC* gene and specimens were attributed a resistant or susceptible (wild-type sequence or synonymous mutations) genotype.^21–23^

### Statistical analysis

We undertook a descriptive statistical analysis to examine the proportion of *M. genitalium* cases with genetic determinants of AMR and the participant characteristics associated with AMR. Exact 95% confidence intervals were calculated, and proportions compared for specimens with genetic determinants of macrolide resistance by sociodemographic, behavioural, and clinical characteristics using two-sided Fisher’s exact tests.

## Results

Of the available specimens that previously tested positive for *M. genitalium*, 94% (66/70) were re-confirmed as positive for *M. genitalium*. Sequencing was successful in 85% (56/66) for 23S rRNA and 92% (61/66) for *parC* genes.

Mutations associated with macrolide resistance, at nucleotide position 2058 or 2059 of the 23S rRNA gene (*Escherichia coli* numbering), were detected in 9/56 (16.1%; 95% CI 8.6% to 27.8%) specimens, with the A2058G mutation most common (n=7), followed by A2059G (n=1) and A2059C (n=1) (table 1). *parC* gene mutations associated with fluoroquinolone resistance (D87N and D87Y)^23 24^ were detected in 2/61 (3.3%; 0.9%–11.2%) specimens. For these two specimens, the 23S rRNA gene sequence was wild-type (n=1) or did not amplify (n=1).

**Table 1:** Wild-Type and antimicrobial resistance-conferring mutations in 23S rRNA and parC genes in *Mycoplasma genitalium* positive specimens from the general British population

23S rRNA gene	No. (n=56)	% (95% confidence intervals)
Wild-type (S)	47	83.9 (72.2 to 91.3)
Mutation detected (R)	9	16.1 (8.6 to 27.8)
A2058G	*7*	
A2059C	*1*	
A2059G	*1*	

parC gene	No. (n=61)	% (95% confidence intervals)
Wild-type (S)*	59	96.7 (88.9 to 99.1)
Mutation detected (R)	2	3.3 (0.9 to 11.2)
D87N	*1*	
D87Y	*1*	

*includes isolates with wild-type sequence (n=56) and synonymous mutations (n=4).

R, resistant; S, susceptible.

Specimens with mutations associated with macrolide resistance were more likely to come from participants reporting a history of diagnosed STIs or sexual health clinic attendance (table 2). Participants reporting any STI diagnoses in the past 5 years were more likely to have macrolide resistant *M. genitalium* than those without a STI diagnosis (44.4% (18.9%–73.3%) vs 10.6% (4.6%–22.6%); p*=*0.029). Similarly, those reporting visiting a sexual health clinic in the past year were more likely to have macrolide resistant *M. genitalium* than those not attending a clinic in the past year (43.8% (23.1%–66.8%) vs 5.0% (1.4%–16.5%); p=0.001). A higher proportion of participants reporting two or more sexual partners in the past year had *M. genitalium* with genotypic macrolide resistance when compared with participants reporting zero or one partner (25.0% (13.3%–42.1%) vs 4.2% (0.7%–20.3%); p=0.063).

**Table 2:** Sociodemographic, behavioural, and clinical risk factors for macrolide resistance-conferring mutations in the 23S rRNA gene in *Mycoplasma genitalium* specimens from a sexually-active probability sample of the general British population

	*M. genitalium* with resistance-conferring mutation			Susceptible *M. genitalium*			Total	P value
	No.	%	95% CI	No		95% CI	No.	
	9	16.1	8.7 to 27.8	47	83.9	72.2 to 91.3	56	
Sex								
Male	3	14.3	5.0 to 34.6	18	85.7	65.4 to 95.0	21	1.000
Female	6	17.1	8.1 to 32.7	29	82.9	67.3 to 91.9	35	
Age (years)								
16–24	5	22.7	10.1 to 43.4	17	77.3	56.6 to 89.9	22	0.479
25–34	4	14.8	5.9 to 32.5	23	85.2	67.5 to 94.1	27	
35–44	0	0	–	7	100.0	64.6 to 100	7	
Ethnic group*								
White	7	15.6	7.8 to 28.8	38	84.4	71.2 to 92.3	45	0.611
Black/Black British	2	25.0	7.2 to 59.1	6	75.0	40.9 to 92.9	8	
No. sexual partners, past year**†**								
2+	8	25.0	13.3 to 42.1	24	75.0	57.9 to 86.8	32	0.063
0–1	1	4.2	0.7 to 20.3	23	95.8	79.8 to 99.3	24	
No. new sexual partners, past year †								
1+	8	24.2	12.8 to 41.0	25	75.7	59.0 to 87.2	33	0.067
0	1	4.4	0.8 to 21.0	22	95.7	79.0 to 99.2	23	
Unsafe sex, past year ‡								
Yes	3	21.4	7.6 to 47.6	11	78.6	52.4 to 92.4	14	0.678
No	6	14.6	6.9 to 28.4	35	85.4	72.6 to 93.1	41	
STI symptoms, past month§								
Yes	0	0	–	12	100.0	75.8 to 100	12	0.180
No/not mentioned	9	20.5	11.2 to 34.5	35	79.5	65.5 to 88.9	44	
Diagnosed with any STI, past 5 years								
Yes	4	44.4	18.9 to 73.3	5	55.6	26.7 to 81.1	9	0.029
No	5	10.6	4.6 to 22.6	42	89.4	77.4 to 95.4	47	
Sexual health clinic attendance								
Yes	9	24.3	13.4 to 40.1	28	75.7	59.9 to 86.6	37	0.021
In the last year	7	43.8	23.1 to 66.8	9	56.3	33.2 to 76.9	16	0.001
1+years ago	2	12.5	3.5 to 36.0	19	90.5	71.1 to 97.4	21	
No	0	0	–	19	100.0	83.2 to 100	19	

*Small numbers of participants in other ethnic groups prevented analysis.

†Includes both opposite-sex and same-sex partners.

‡Had sex with at least two partners in the past year and did not use a condom during this timeframe; one participant did not respond to this question.

§STI symptoms included: pain or increased frequency of urination, presence of genital warts or ulcers, penile discharge or abnormal/odorous vaginal discharge, painful testicles, vaginal pain during sex, bleeding between periods or after sex and lower abdominal/pelvic pain.

All four participants with macrolide-resistant specimens and STI diagnoses (table 2) had been diagnosed with *C. trachomatis* in the preceding 5 years, two of whom had *Neisseria gonorrhoeae*, genital warts and non-specific urethritis (NSU), and one of whom had *Treponema pallidum*. Of the participants with macrolide-susceptible isolates and STI diagnoses (n=5) (table 2), three had been diagnosed with *C. trachomatis*, one with genital warts and one with *Trichomonas vaginalis*.

We observed that none of 11 participants with *M. genitalium* AMR mutations (in 23 rRNA or *parC* genes) reported any symptoms in the past month. Furthermore, these 11 participants all reported sexual health clinic attendance in the past 5 years (seven within the past year). Small numbers prevented further epidemiological characterisation of specimens with mutations in the *parC* gene.

## Discussion

To our knowledge, this is the first probability sample study representative of the general population to assess the prevalence of mutations associated with AMR in *M. genitalium* specimens at a national level. Among *M. genitalium* positive specimens collected in 2010–12, we observed genetic determinants of macrolide resistance in one in six (16.1%) specimens and determinants of fluoroquinolone resistance in one in thirty (3.3%). Specimens with macrolide resistance were more likely to come from participants reporting a history of diagnosed bacterial STIs or previous clinic attendance. It was not possible to determine whether the observed prevalence of AMR is attributable to sexual transmission of resistant *M. genitalium* strains or the emergence of de novo resistance.

Study strengths include the high proportion of specimens with sequencing data and the collection of specimens from the general population, which is important because previous studies of *M. genitalium* AMR have mainly used convenience samples, often drawn from clinic or STI-diagnosed populations, where selection bias may lead to overestimating the scale of the problem. One clinical study collected data at a similar time to Natsal-3 (2010–12) in the UK and provides relevant data for comparison. Pond *et al* undertook an observational study to assess the proportion with genetic AMR determinants in men presenting with urethritis to a London sexual health clinic in 2011. Nine of 22 (41%) cases with *M. genitalium* had 23S rRNA gene mutations associated with macrolide resistance, and one case had a *parC* mutation associated with fluoroquinolone resistance. As might be expected, compared with this clinical study, the prevalence with resistance detected was lower in specimens collected from the general population in Natsal. Our study also provides an opportunity to assess AMR in symptomatic and asymptomatic people with *M. genitalium* in the general population. We expect the high levels of AMR found in Britain to have increased since the specimens were collected given high levels reported for other countries at later times, and we will update findings for the UK in 2022 through the Natsal-4 study.^25^

Potential limitations include that specimens were stored for up to 6 years before re-confirmation and AMR testing, with the potential for specimen degradation, which may explain instances of assay failure. Numerous mutations have been reported in the *parC* gene highlighting the variability of this region, and we have inferred phenotype from genotype. However, in the present study we only report on 23S rRNA or *parC* gene mutations that have been associated with azithromycin or moxifloxacin resistance. The challenge for the microbiological community will be to improve culture capacity and sequencing directly from clinical specimens to better understand how other 23S rRNA gene or *parC* mutations are associated with genotype, phenotype and treatment failures. Furthermore, widely implementing test of cure, genetic AMR testing and surveillance of treatment failures will improve understanding of the correlation between genotype and clinical outcome. Finally, small numbers of *M. genitalium* positive specimens, even within such a large study, meant there was not sufficient statistical power to rule out associations being present where none were observed.

It was striking that none of the specimens with an AMR-conferring genotype were from participants reporting STI symptoms. Under the current UK treatment guidelines, asymptomatic patients are not recommended for *M. genitalium* testing except sexual contacts, and it is unlikely that any of these participants would have been tested. We need better understanding about the implications of asymptomatic infection, which might resolve with limited sequelae. The current approach might need rethinking if asymptomatic infections are found to be an important reservoir for AMR and/or a source of infection and disease. Given that all participants with AMR specimens had attended clinics in the past 5 years (most in the last year), modelling studies might be undertaken to investigate the cost effectiveness of routinely testing asymptomatic sexual health clinic attendees (ie, clinic-based screening) for *M. genitalium* and whether this approach might assist in the control of *M. genitalium* and associated AMR.

In conclusion, our data support *M. genitalium* detection strategies that include pre-treatment macrolide resistance testing to guide therapy, with the use of moxifloxacin treatment where macrolide resistance is identified. We recognise that availability of commercial validated and quality-assured assays for macrolide resistance detection may vary (and there are currently no commercial assays for detection of fluoroquinolone resistance), and even diagnostic testing is not yet widely obtainable in most countries. However, a promising approach has been described in Australia, where doxycycline therapy was followed by macrolide resistance testing to guide treatment. In this study, where azithromycin was given only to macrolide-susceptible cases and sitafloxacin therapy was used for macrolide-resistant cases,>92% of *M. genitalium* infections were shown to be cured.^26^ Our data highlight the significant public health challenges in the control of *M. genitalium,* including an urgent need for evidence-based third line treatments, such as pristinamycin, minocycline or new antimicrobials such as lefamulin, gepotidacin, solithromycin or zoliflodacin,^27–30^ and the need for systematic national and international surveillance of AMR and treatment failure to inform treatment guidelines.

**Key messages**

- We investigated the prevalence of antimicrobial resistance (AMR)-conferring mutations in *M. genitalium* among the sexually-active British general population
- Macrolide resistance-conferring mutations were detected in 16.1% (95%CI: 8.6% to 27.8%) and fluoroquinolone resistance-conferring mutations were detected in 3.3% (0.9%–11.2%) of specimens with *M. genitalium* detected
- Specimens with macrolide resistance were more likely to come from participants reporting a history of diagnosed bacterial STIs or recent sexual health clinic attendance.
- All participants with AMR-conferring mutations had attended sexual health clinics, but none reported recent symptoms
- These data highlight challenges in *M. genitalium* management and control
